# Supplementary material for: Machine Learning Accelerates Crystallization for Structure Determination
Source: Angew Chem Int Ed Engl. 2026 May 4;65(25):e1218503. doi: 10.1002/anie.1218503 (PMC13266958; doi:10.1002/anie.1218503)

checkCIF/PLATON report

Structure factors have been supplied for datablock(s) lcz026

THIS REPORT IS FOR GUIDANCE ONLY. IF USED AS PART OF A REVIEW PROCEDURE FOR PUBLICATION, IT SHOULD NOT REPLACE THE EXPERTISE OF AN EXPERIENCED CRYSTALLOGRAPHIC REFEREE.

No syntax errors found. CIF dictionary Interpreting this report

Datablock: lcz026

Bond precision:	C-C = 0.0181 A		Wavelength=1.54184
Cell:	a=24.8797(2)	b=22.3473(1)	c=25.4100(2)
	alpha=90	beta=110.261(1)	gamma=90
Temperature:	100 K		

	Calculated	Reported
Volume	13253.65(18)	13253.65(18)
Space group	P 21/c	P 1 21/c 1
Hall group	-P 2ybc	-P 2ybc
Moiety formula	C37 H27 Ag3 F18 N6 O4, C26 H15 Ag3 F18 N6 O2, 2(C15 H3 Ag3 F18	C15 H3 Ag3 F18 N6, C11 H12 O2
Sum formula	C104 H60 Ag12 F72 N24 O8	C26 H15 Ag3 F18 N6 O2
Mr	4436.20	1109.05
Dx,g cm-3	2.223	2.223
Z	4	16
Mu (mm-1)	15.399	15.399
F000	8512.0	8512.0
F000’	8553.20	
h,k,lmax	31,28,32	31,28,31
Nref	28548	27284
Tmin,Tmax	0.162,0.158	0.193,1.000
Tmin’	0.045	

```
Correction method= # Reported T Limits: Tmin=0.193 Tmax=1.000
AbsCorr = MULTI-SCAN

Data completeness= 0.956            Theta(max)= 78.545

R(reflections)= 0.0766( 23028)      wR2(reflections)= 0.2061( 27284)

S = 1.067                  Npar= 2069
```

The following ALERTS were generated. Each ALERT has the format
test-name_ALERT_alert-type_alert-level.
Click on the hyperlinks for more details of the test.

Alert level C

```
PLAT041_ALERT_1_C Calc. and Reported SumFormula    Strings  Differ     Please Check
              Calc: C104 H60 Ag12 F72 N24 O8
              Rep.: C26 H15 Ag3 F18 N6 O2
PLAT042_ALERT_1_C Calc. and Reported MoietyFormula Strings  Differ     Please Check
              Calc: C37 H27 Ag3 F18 N6 O4, C26 H15 Ag3 F18 N6 O2,
                    2(C15 H3 Ag3 F18 N6), C11 H12 O2
              Rep.: C15 H3 Ag3 F18 N6, C11 H12 O2
PLAT213_ALERT_2_C Atom F1A            has ADP max/min Ratio .....        3.1 prolat
PLAT213_ALERT_2_C Atom F02C           has ADP max/min Ratio .....        3.6 prolat
PLAT213_ALERT_2_C Atom N015           has ADP max/min Ratio .....        3.1 oblate
PLAT220_ALERT_2_C NonSolvent   Resd 1  C   Ueq(max)/Ueq(min) Range       3.7 Ratio
PLAT230_ALERT_2_C Hirshfeld Test Diff for    C05B     --C05K     .       5.7 s.u.
PLAT230_ALERT_2_C Hirshfeld Test Diff for    F02H     --C046     .       5.1 s.u.
PLAT230_ALERT_2_C Hirshfeld Test Diff for    F02K     --C041     .       6.1 s.u.
PLAT230_ALERT_2_C Hirshfeld Test Diff for    F026     --C03X     .       6.3 s.u.
PLAT230_ALERT_2_C Hirshfeld Test Diff for    F039     --C052     .       5.1 s.u.
PLAT230_ALERT_2_C Hirshfeld Test Diff for    F02Z     --C04X     .       6.1 s.u.
PLAT230_ALERT_2_C Hirshfeld Test Diff for    F03L     --C04X     .       5.6 s.u.
PLAT234_ALERT_4_C Large Hirshfeld Difference F032     --C05E     .      0.20 Ang.
PLAT234_ALERT_4_C Large Hirshfeld Difference F02C     --C04F     .      0.16 Ang.
PLAT234_ALERT_4_C Large Hirshfeld Difference F8       --C05T     .      0.18 Ang.
PLAT234_ALERT_4_C Large Hirshfeld Difference F02J     --C05N     .      0.17 Ang.
PLAT234_ALERT_4_C Large Hirshfeld Difference F02Q     --C03X     .      0.18 Ang.
PLAT234_ALERT_4_C Large Hirshfeld Difference F02S     --C052     .      0.22 Ang.
PLAT234_ALERT_4_C Large Hirshfeld Difference F033     --C057     .      0.17 Ang.
PLAT234_ALERT_4_C Large Hirshfeld Difference F04Q     --C03X     .      0.19 Ang.
PLAT234_ALERT_4_C Large Hirshfeld Difference F05A     --C052     .      0.19 Ang.
PLAT234_ALERT_4_C Large Hirshfeld Difference C04G     --C05T     .      0.16 Ang.
PLAT234_ALERT_4_C Large Hirshfeld Difference F02F     --C04T     .      0.20 Ang.
PLAT234_ALERT_4_C Large Hirshfeld Difference F03S     --C04T     .      0.21 Ang.
PLAT234_ALERT_4_C Large Hirshfeld Difference F04Y     --C04X     .      0.19 Ang.
PLAT234_ALERT_4_C Large Hirshfeld Difference O05D     --C05W     .      0.17 Ang.
PLAT234_ALERT_4_C Large Hirshfeld Difference C05P     --C062     .      0.19 Ang.
PLAT234_ALERT_4_C Large Hirshfeld Difference C05R     --C05Y     .      0.19 Ang.
PLAT241_ALERT_2_C High   'MainMol' Ueq as Compared to Neighbors of      O018 Check
PLAT241_ALERT_2_C High   'MainMol' Ueq as Compared to Neighbors of      O042 Check
PLAT241_ALERT_2_C High   'MainMol' Ueq as Compared to Neighbors of      C05B Check
PLAT241_ALERT_2_C High   'MainMol' Ueq as Compared to Neighbors of      C067 Check
PLAT241_ALERT_2_C High   'MainMol' Ueq as Compared to Neighbors of      O01X Check
```

```
PLAT242_ALERT_2_C Low    'MainMol' Ueq as Compared to Neighbors of       Ag05 Check
PLAT242_ALERT_2_C Low    'MainMol' Ueq as Compared to Neighbors of       C049 Check
PLAT242_ALERT_2_C Low    'MainMol' Ueq as Compared to Neighbors of       C04R Check
PLAT242_ALERT_2_C Low    'MainMol' Ueq as Compared to Neighbors of       C05S Check
PLAT342_ALERT_3_C Low Bond Precision on  C-C Bonds ..............    0.01811 Ang.
PLAT369_ALERT_2_C Long   C(sp2)-C(sp2) Bond  C05M     - C060     .       1.56 Ang.
PLAT790_ALERT_4_C Centre of Gravity not Within Unit-Cell: Resd.  #          1 Note
              C37 H27 Ag3 F18 N6 O4
PLAT906_ALERT_3_C Large K Value in the Analysis of Variance ......     10.103 Check
PLAT906_ALERT_3_C Large K Value in the Analysis of Variance ......      2.318 Check
PLAT911_ALERT_3_C Missing FCF Refl Between Thmin & STh/L=    0.600         92 Report
                0  2  0,   0 12  0,   4  0  0,  -6  2  1,  -4  1  2,  -3  0  2,
               -1  0  2,   0  1  2,  24  7  2,  -9 17  3,  -6 18  3,  22  8  3,
              -10 10  6,  -9  7  6,  -8  9  6,  -1  0  8, -11  8  9, -10  9  9,
                5  7  9, -10  8 10,  -6 11 10,   5  6 10,  -9  6 11, -20 17 13,
              -17 21 13,  -8  7 13,  20  0 14,  20  1 14,  20  2 14,   8  1 15,
                   (   62 More Missing: see the .ckf listing file)
PLAT971_ALERT_2_C Check Calcd Resid. Dens.  0.95Ang From F03L           2.39 eA-3
PLAT971_ALERT_2_C Check Calcd Resid. Dens.  1.06Ang From C05K           2.18 eA-3
PLAT971_ALERT_2_C Check Calcd Resid. Dens.  0.80Ang From F026           2.08 eA-3
PLAT971_ALERT_2_C Check Calcd Resid. Dens.  1.05Ang From F02F           2.03 eA-3
PLAT971_ALERT_2_C Check Calcd Resid. Dens.  1.05Ang From F02F           2.03 eA-3
PLAT971_ALERT_2_C Check Calcd Resid. Dens.  1.02Ang From F03S           2.02 eA-3
PLAT971_ALERT_2_C Check Calcd Resid. Dens.  0.80Ang From Ag04           2.01 eA-3
PLAT971_ALERT_2_C Check Calcd Resid. Dens.  0.57Ang From F02K           2.00 eA-3
PLAT971_ALERT_2_C Check Calcd Resid. Dens.  0.89Ang From F04Q           1.99 eA-3
PLAT971_ALERT_2_C Check Calcd Resid. Dens.  0.81Ang From F02K           1.99 eA-3
PLAT971_ALERT_2_C Check Calcd Resid. Dens.  0.79Ang From F02S           1.95 eA-3
PLAT971_ALERT_2_C Check Calcd Resid. Dens.  0.74Ang From F02Q           1.94 eA-3
PLAT971_ALERT_2_C Check Calcd Resid. Dens.  1.04Ang From F02Z           1.94 eA-3
PLAT971_ALERT_2_C Check Calcd Resid. Dens.  0.86Ang From Ag0A           1.85 eA-3
PLAT971_ALERT_2_C Check Calcd Resid. Dens.  0.81Ang From C05W           1.82 eA-3
PLAT971_ALERT_2_C Check Calcd Resid. Dens.  0.76Ang From C061           1.81 eA-3
PLAT971_ALERT_2_C Check Calcd Resid. Dens.  0.70Ang From F02J           1.80 eA-3
PLAT971_ALERT_2_C Check Calcd Resid. Dens.  1.54Ang From F033           1.80 eA-3
PLAT971_ALERT_2_C Check Calcd Resid. Dens.  0.69Ang From F02Q           1.77 eA-3
PLAT971_ALERT_2_C Check Calcd Resid. Dens.  0.82Ang From Ag05           1.77 eA-3
PLAT971_ALERT_2_C Check Calcd Resid. Dens.  0.74Ang From Ag0C           1.76 eA-3
PLAT971_ALERT_2_C Check Calcd Resid. Dens.  0.83Ang From Ag01           1.76 eA-3
PLAT971_ALERT_2_C Check Calcd Resid. Dens.  0.79Ang From Ag08           1.75 eA-3
PLAT971_ALERT_2_C Check Calcd Resid. Dens.  0.83Ang From Ag02           1.73 eA-3
PLAT971_ALERT_2_C Check Calcd Resid. Dens.  0.71Ang From F02F           1.73 eA-3
PLAT972_ALERT_2_C Check Calcd Resid. Dens.  0.68Ang From F02Z          -1.66 eA-3
PLAT972_ALERT_2_C Check Calcd Resid. Dens.  0.58Ang From F02K          -1.57 eA-3
PLAT972_ALERT_2_C Check Calcd Resid. Dens.  0.62Ang From C061          -1.53 eA-3
PLAT972_ALERT_2_C Check Calcd Resid. Dens.  0.54Ang From F02K          -1.53 eA-3
PLAT977_ALERT_2_C Check Negative Difference Density on H02V      .      -0.35 eA-3
PLAT977_ALERT_2_C Check Negative Difference Density on H03A      .      -0.36 eA-3
PLAT977_ALERT_2_C Check Negative Difference Density on H05Q      .      -0.35 eA-3
PLAT977_ALERT_2_C Check Negative Difference Density on H05V      .      -0.34 eA-3
```

Alert level G

```
PLAT002_ALERT_2_G Number of Distance or Angle Restraints on AtSite         21 Note
PLAT003_ALERT_2_G Number of Uiso or U(i,j)  Restrained non-H-Atoms         45 Report
PLAT045_ALERT_1_G Calculated and Reported Z Differ by a Factor ...      0.250 Check
PLAT083_ALERT_2_G SHELXL Second Parameter in WGHT  Unusually Large     247.75 Why ?
```

```
PLAT142_ALERT_4_G s.u. on b - Axis Small or Missing .............    0.00010 Ang.
PLAT143_ALERT_4_G s.u. on c - Axis Small or Missing .............    0.00020 Ang.
PLAT176_ALERT_4_G The CIF-Embedded .res File Contains SADI Records         9 Report
PLAT177_ALERT_4_G The CIF-Embedded .res File Contains DELU Records         6 Report
PLAT178_ALERT_4_G The CIF-Embedded .res File Contains SIMU Records         1 Report
PLAT186_ALERT_4_G The CIF-Embedded .res File Contains ISOR Records         9 Report
PLAT188_ALERT_3_G A Non-default SIMU Restraint Value has been used    0.0200 Report
PLAT192_ALERT_3_G A Non-default DELU Restraint Value for SecondPar    0.0200 Report
PLAT192_ALERT_3_G A Non-default DELU Restraint Value for SecondPar    0.0200 Report
PLAT192_ALERT_3_G A Non-default DELU Restraint Value for SecondPar    0.0200 Report
PLAT192_ALERT_3_G A Non-default DELU Restraint Value for SecondPar    0.0200 Report
PLAT192_ALERT_3_G A Non-default DELU Restraint Value for SecondPar    0.0200 Report
PLAT192_ALERT_3_G A Non-default DELU Restraint Value for SecondPar    0.0200 Report
PLAT230_ALERT_2_G Hirshfeld Test Diff for    F2       --C4       .       5.8 s.u.
PLAT242_ALERT_2_G Low    'MainMol' Ueq as Compared to Neighbors of        C4 Check
PLAT242_ALERT_2_G Low    'MainMol' Ueq as Compared to Neighbors of      C02O Check
PLAT242_ALERT_2_G Low    'MainMol' Ueq as Compared to Neighbors of      C04F Check
PLAT242_ALERT_2_G Low    'MainMol' Ueq as Compared to Neighbors of      C04I Check
PLAT242_ALERT_2_G Low    'MainMol' Ueq as Compared to Neighbors of      C050 Check
PLAT242_ALERT_2_G Low    'MainMol' Ueq as Compared to Neighbors of      C05E Check
PLAT242_ALERT_2_G Low    'MainMol' Ueq as Compared to Neighbors of      C03Q Check
PLAT242_ALERT_2_G Low    'MainMol' Ueq as Compared to Neighbors of      C041 Check
PLAT242_ALERT_2_G Low    'MainMol' Ueq as Compared to Neighbors of      C046 Check
PLAT242_ALERT_2_G Low    'MainMol' Ueq as Compared to Neighbors of      C04E Check
PLAT242_ALERT_2_G Low    'MainMol' Ueq as Compared to Neighbors of      C04Z Check
PLAT242_ALERT_2_G Low    'MainMol' Ueq as Compared to Neighbors of      C05C Check
PLAT242_ALERT_2_G Low    'MainMol' Ueq as Compared to Neighbors of      C03N Check
PLAT242_ALERT_2_G Low    'MainMol' Ueq as Compared to Neighbors of      C03X Check
PLAT242_ALERT_2_G Low    'MainMol' Ueq as Compared to Neighbors of      C052 Check
PLAT242_ALERT_2_G Low    'MainMol' Ueq as Compared to Neighbors of      C057 Check
PLAT242_ALERT_2_G Low    'MainMol' Ueq as Compared to Neighbors of      C05N Check
PLAT242_ALERT_2_G Low    'MainMol' Ueq as Compared to Neighbors of      C05T Check
PLAT242_ALERT_2_G Low    'MainMol' Ueq as Compared to Neighbors of      C034 Check
PLAT242_ALERT_2_G Low    'MainMol' Ueq as Compared to Neighbors of      C04J Check
PLAT242_ALERT_2_G Low    'MainMol' Ueq as Compared to Neighbors of      C04L Check
PLAT242_ALERT_2_G Low    'MainMol' Ueq as Compared to Neighbors of      C04O Check
PLAT242_ALERT_2_G Low    'MainMol' Ueq as Compared to Neighbors of      C04T Check
PLAT242_ALERT_2_G Low    'MainMol' Ueq as Compared to Neighbors of      C04X Check
PLAT301_ALERT_3_G Main Residue  Disorder .............(Resd    1)         9% Note
PLAT302_ALERT_4_G Anion/Solvent/Minor-Residue Disorder (Resd    3)         7% Note
PLAT335_ALERT_2_G Check Large C6 Ring C-C Range C049     -C05B           0.25 Ang.
PLAT335_ALERT_2_G Check Large C6 Ring C-C Range C05F     -C05R           0.21 Ang.
PLAT434_ALERT_2_G Short Inter HL..HL Contact F00E     ..F00Q     .       2.79 Ang.
                                                    x,y,z  =      1_555 Check
PLAT434_ALERT_2_G Short Inter HL..HL Contact F00U     ..F032     .       2.75 Ang.
                                        2-x,-1/2+y,1/2-z  =      2_745 Check
PLAT434_ALERT_2_G Short Inter HL..HL Contact F01G     ..F02E     .       2.79 Ang.
                                          x,3/2-y,-1/2+z  =      4_575 Check
PLAT434_ALERT_2_G Short Inter HL..HL Contact F024     ..F3A      .       2.54 Ang.
                                              2-x,2-y,-z  =      3_775 Check
PLAT434_ALERT_2_G Short Inter HL..HL Contact F02E     ..F036     .       2.70 Ang.
                                           x,3/2-y,1/2+z  =      4_576 Check
PLAT720_ALERT_4_G Number of Unusual/Non-Standard Labels .........        277 Note
              Ag01    Ag02    Ag03    Ag04    Ag05    Ag06    Ag07    Ag08
              Ag09    Ag0A    Ag0B    Ag0C    F00D    F00E    F00F    F00G
              F00H    F00I    F00J    F00K    F00L    F00M    F00N    N00O
              F00P    F00Q    F00R    F00S    N00T    F00U    F00V    F00W
```

F00X	N00Y	F00Z	N010	N011	N012	F013	F014
N015	N016	N017	O018	N019	N01A	F01B	F01C
F01D	F01E	N01F	F01G	N01H	F01I	F01J	F01K
F01L	N01M	N01N	F01O	N01P	N01Q	N01R	N01S
N01T	N01U	O01V	F01W	O01X	N01Y	N01Z	N020
O021	F022	F023	F024	F025	F026	F027	F028
F029	F02A	F02B	F02C	F02D	F02E	F02F	F02G
F02H	C02I	H02I	F02J	F02K	C02L	C02M	C02N
C02O	C02P	H02P	F02Q	F02R	F02S	F02T	C02U
H02U	C02V	H02V	C02W	F02X	C02Y	F02Z	C030
C031	H031	F032	F033	C034	O035	F036	C038
F039	C03A	C03B	C03C	H03C	C03D	C03E	H03E
F03F	C03G	C03H	H03A	H03B	C03I	O03J	C03K
H03K	F03L	C03M	H03M	C03N	C03O	C03P	C03Q
C03R	H03R	F03S	C03T	C03U	C03V	H03V	C03W
H03W	C03X	C03Y	C03Z	H03Z	C040	H04A	H04B
C041	O042	C044	C045	C046	C047	H04G	H04H
C048	H048	C049	H049	C04A	C04B	C04D	C04E
C04F	C04G	C04H	H04C	H04D	C04I	C04J	C04K
C04L	C04M	C04N	H04N	C04O	C04P	F04Q	C04R
C04S	H04S	C04T	C04U	C04V	C04W	H04E	H04F
C04X	F04Y	C04Z	C050	C051	C052	C053	H05D
H05E	C054	C055	C056	C057	C058	H058	C059
F05A	C05B	H05P	C05C	O05D	C05E	C05F	C05G
H05G	C05H	C05K	H05Q	C05L	H05L	C05M	H05M
C05N	C05O	H05F	H05H	H05I	C05P	H05J	H05K
C05Q	H05A	H05B	H05C	C05R	C05S	H05R	H05S
C05T	C05V	H05T	H05U	C05W	C05X	H05V	H05W
H05X	C05Y	C05Z	H05N	H05O	C060	H060	C061
C062	H06A	H06B	C063	H06C	H06D	H06E	F064
F065	C067	H06F	H06G	F0			

PLAT860_ALERT_3_G Number of Least-Squares Restraints .... 261 Note
PLAT883_ALERT_1_G Absent Datum for _atom_sites_solution_primary .. Please Do !
PLAT910_ALERT_3_G Missing FCF Reflection(s) Below Theta(Min)[Deg]= 2.92 Note
1 0 0, 1 1 0, 0 1 1,
PLAT912_ALERT_4_G Missing # of FCF Reflections Above STh/L= 0.600 1138 Note
PLAT913_ALERT_3_G Missing # of Very Strong Reflections in FCF .... 3 Note
4 0 0, -6 2 1, -1 0 8,
PLAT933_ALERT_2_G Number of HKL-OMIT Records in Embedded .res File 61 Note
-7 1 31, -7 3 31, -5 0 30, -4 1 29, -4 4 30, -4 5 30,
-4 6 30, -3 0 28, -3 5 29, -3 6 29, -3 7 29, -3 9 29,
-2 1 27, -2 4 29, -2 5 29, -2 6 29, -2 7 29, -2 8 29,
-1 1 27, -1 2 28, -1 3 28, -1 4 28, -1 5 28, -1 6 28,
0 0 26, 0 3 28, 0 4 27, 0 4 28, 0 6 28, 0 7 28,
1 0 26, 1 1 25, 1 2 27, 1 4 27, 2 2 23, 2 2 24,
3 0 24, 3 1 23, 3 1 24, 3 2 22, 3 2 23, 4 1 22,
4 2 22, 4 13 21, 5 12 21, 6 12 18, 6 12 20, 8 1 16,
8 11 18, 9 10 18,
PLAT941_ALERT_3_G Average HKL Measurement Multiplicity .... 2.6 Low
PLAT969_ALERT_5_G The 'Henn et al.' R-Factor-gap value .... 4.050 Note
Predicted wR2: Based on SigI**2 5.09 or SHELX Weight 19.32
PLAT978_ALERT_2_G Number C-C Bonds with Positive Residual Density. 0 Info

---

0 **ALERT level A** = Most likely a serious problem - resolve or explain
0 **ALERT level B** = A potentially serious problem, consider carefully
77 **ALERT level C** = Check. Ensure it is not caused by an omission or oversight

```
  61 ALERT level G = General information/check it is not something unexpected

   4 ALERT type 1 CIF construction/syntax error, inconsistent or missing data
  91 ALERT type 2 Indicator that the structure model may be wrong or deficient
  16 ALERT type 3 Indicator that the structure quality may be low
  26 ALERT type 4 Improvement, methodology, query or suggestion
   1 ALERT type 5 Informative message, check
```

Validation response form

Please find below a validation response form (VRF) that can be filled in and pasted into your CIF.

```
# start Validation Reply Form
_vrf_PLAT041_lcz026
;
PROBLEM: Calc. and Reported SumFormula    Strings  Differ     Please Check
RESPONSE: ...
;
_vrf_PLAT042_lcz026
;
PROBLEM: Calc. and Reported MoietyFormula Strings  Differ     Please Check
RESPONSE: ...
;
_vrf_PLAT213_lcz026
;
PROBLEM: Atom F1A             has ADP max/min Ratio .....        3.1 prolat
RESPONSE: ...
;
_vrf_PLAT220_lcz026
;
PROBLEM: NonSolvent   Resd 1  C   Ueq(max)/Ueq(min) Range        3.7 Ratio
RESPONSE: ...
;
_vrf_PLAT230_lcz026
;
PROBLEM: Hirshfeld Test Diff for    C05B     --C05K     .        5.7 s.u.
RESPONSE: ...
;
_vrf_PLAT234_lcz026
;
PROBLEM: Large Hirshfeld Difference F032     --C05E     .       0.20 Ang.
RESPONSE: ...
;
_vrf_PLAT241_lcz026
;
PROBLEM: High   'MainMol' Ueq as Compared to Neighbors of       O018 Check
RESPONSE: ...
;
_vrf_PLAT242_lcz026
;
PROBLEM: Low    'MainMol' Ueq as Compared to Neighbors of       Ag05 Check
RESPONSE: ...
;
_vrf_PLAT342_lcz026
;
```

```
PROBLEM: Low Bond Precision on  C-C Bonds ..............    0.01811 Ang.
RESPONSE: ...
;
_vrf_PLAT369_lcz026
;
PROBLEM: Long   C(sp2)-C(sp2) Bond  C05M     - C060     .       1.56 Ang.
RESPONSE: ...
;
_vrf_PLAT790_lcz026
;
PROBLEM: Centre of Gravity not Within Unit-Cell: Resd.  #          1 Note
RESPONSE: ...
;
_vrf_PLAT906_lcz026
;
PROBLEM: Large K Value in the Analysis of Variance ......     10.103 Check
RESPONSE: ...
;
_vrf_PLAT911_lcz026
;
PROBLEM: Missing FCF Refl Between Thmin & STh/L=    0.600         92 Report
RESPONSE: ...
;
_vrf_PLAT971_lcz026
;
PROBLEM: Check Calcd Resid. Dens.  0.95Ang From F03L           2.39 eA-3
RESPONSE: ...
;
_vrf_PLAT972_lcz026
;
PROBLEM: Check Calcd Resid. Dens.  0.68Ang From F02Z          -1.66 eA-3
RESPONSE: ...
;
_vrf_PLAT977_lcz026
;
PROBLEM: Check Negative Difference Density on H02V      .      -0.35 eA-3
RESPONSE: ...
;
# end Validation Reply Form
```

It is advisable to attempt to resolve as many as possible of the alerts in all categories. Often the minor alerts point to easily fixed oversights, errors and omissions in your CIF or refinement strategy, so attention to these fine details can be worthwhile. It is up to the individual to critically assess their own results and, if necessary, seek expert advice.

PLATON version of 17/09/2025; check.def file version of 17/09/2025

---

duplicate check

No duplication found

Datablock lcz026 - ellipsoid plot

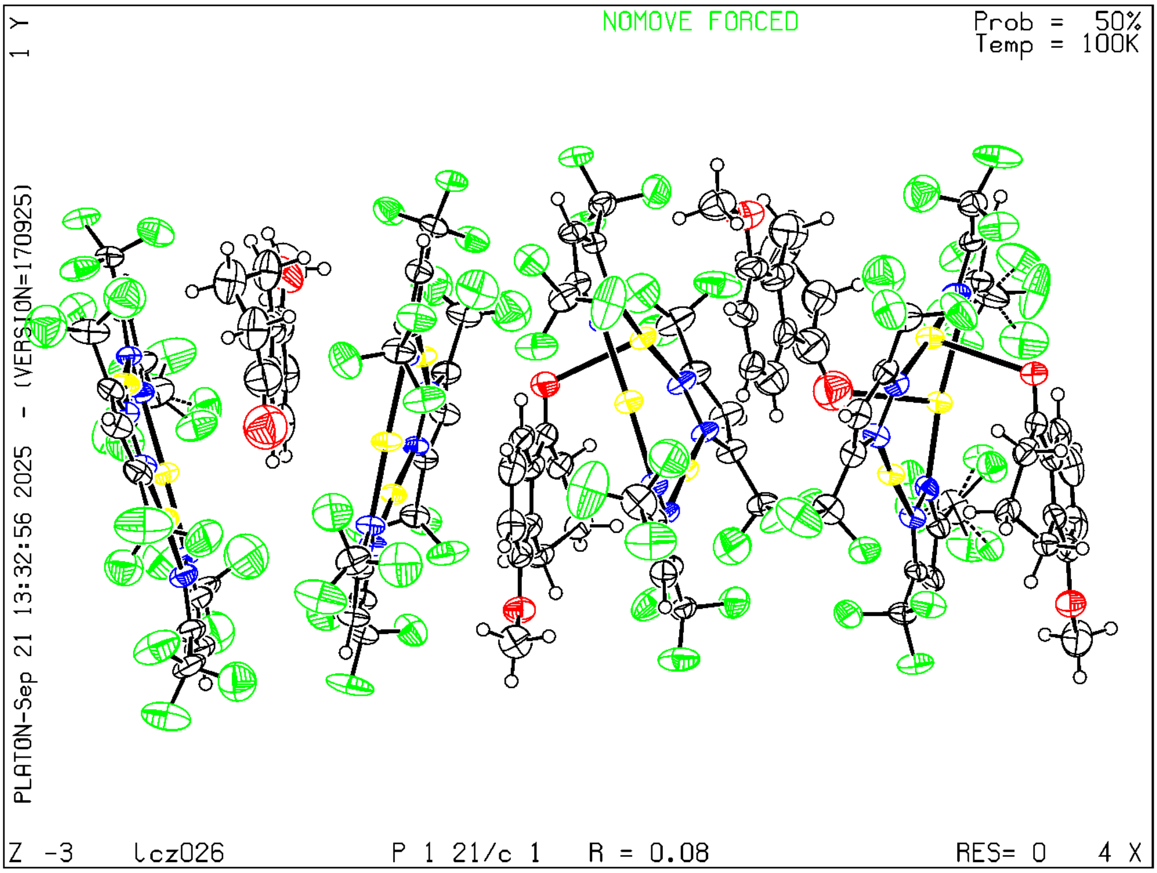

Supplement: Supplementary file 2 — Supporting File 2: anie72462‐sup‐0002‐DataFile.zip. [file ANIE-65-e1218503-s002.zip › supplementary_materials_crystallographic_data submit.zip!/cif_and_checkcif(1-114)/Ag3Pz3_26/Ag3Pz3_26_checkcif.pdf]
